# Correction: Toggle switch residues control allosteric transitions in bacterial adhesins by participating in a concerted repacking of the protein core

**DOI:** 10.1371/journal.ppat.1013925

**Published:** 2026-02-02

**Authors:** Dagmara I. Kisiela, Pearl Magala, Gianluca Interlandi, Laura A. Carlucci, Angelo Ramos, Veronika Tchesnokova, Benjamin Basanta, Vladimir Yarov-Yarovoy, Hovhannes Avagyan, Anahit Hovhannisyan, Wendy E. Thomas, Ronald E. Stenkamp, Rachel E. Klevit, Evgeni V. Sokurenko

The Data Availability statement for this article is incorrect. The correct statement is: All relevant data are within the paper and its Supporting information files.

The hyperlink in S1 Movie is also incorrect. The correct hyperlink is: https://zenodo.org/records/18148857/files/tmd_50ns_34-46ns_40sec_labels.mpg. Please view the correct S1 Movie below.

## Supporting information

S1 Movie Movie showing the TMD trajectory between 34 ns and 46 ns. During this simulation time frame, the flipping of residues L34 (at 4 seconds in the movie) and V35 (at 37 seconds) was observed. L34 and V35 are colored in blue and red, respectively, and their side chains are shown in the stick and ball representation and labeled. Distances between atoms involved in backbone hydrogen bonds between V36 and L107 are indicated by blue dashed lines. Atoms involved in the V36 NH… O L107 hydrogen bond are colored in black while those in the L107 NH… O V36 hydrogen bond are colored in green, respectively.
(DOCX)

## References

[1] Kisiela DI, Magala P, Interlandi G, Carlucci LA, Ramos A, Tchesnokova V, et al. Toggle switch residues control allosteric transitions in bacterial adhesins by participating in a concerted repacking of the protein core. PLoS Pathog. 2021;17(4):e1009440. doi: 10.1371/journal.ppat.1009440 33826682 PMC8064603

