# Supplementary figures and images for: Toggle switch residues control allosteric transitions in bacterial adhesins by participating in a concerted repacking of the protein core
Source: PLoS Pathog. 2021 Apr 7;17(4):e1009440. doi: 10.1371/journal.ppat.1009440 (PMC8064603; doi:10.1371/journal.ppat.1009440)

**A**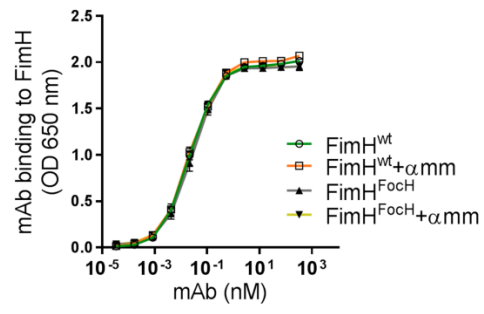**B**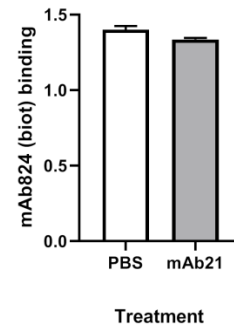**C**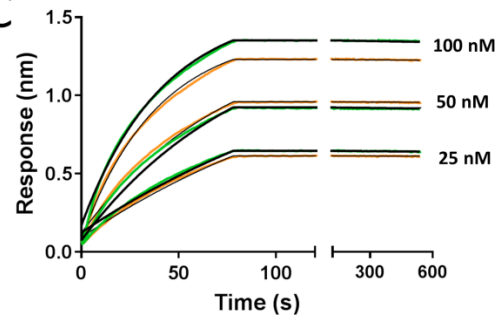

Supplement: S1 Fig — (A) Dose-dependent mAb824 binding in the absence and the presence of 1% αmm to surface-immobilized fimbriae bearing different variant of FimH by ELISA. Data are mean ± SEM, n = 3. (B) Binding of mAb824 (biotinylated) to PBS-treated- and mAb21-complexed FimHwt fimbriae. (C) Binding of FimHwt fimbriae (at 25–100 nM concentrations) to probe-immobilized mAb824 in the absence (green) and presence (orange) of 1% αmm measured by biolayer interferometry (BLI). Kinetic data were globally fitted (black lines) to a 1:1 Langmuir binding model (Prism 6, GraphPad) and obtained kinetic parameters are reported in the text. Data from one representative experiment are shown. (PDF) [file ppat.1009440.s001.pdf]

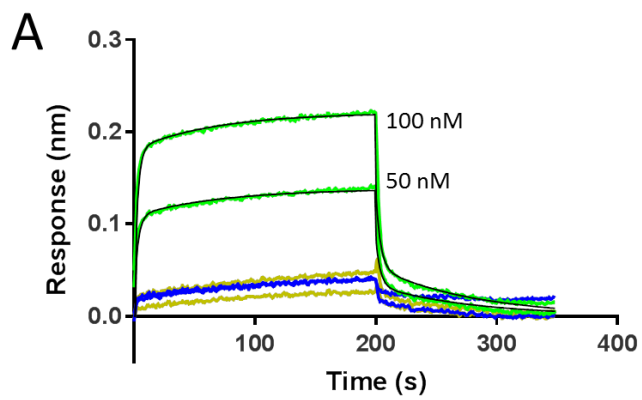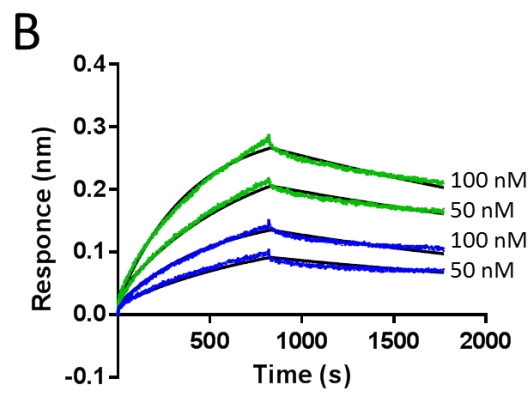

Supplement: S2 Fig — Kinetics of FimHwt (A) and FimHFocH (B) fimbriae binding to ribonuclease B (RNaseB) measured by biolayer interferometry. Recorded sensorgrams show binding of isolated fimbriae to RNaseB (biotinylated) captured on a streptavidin-coated probe. Binding of fimbriae (at 50 nM and 100 nM concentrations) in the absence (green), and the presence of Fab824 (blue) or αmm (yellow). The binding data were globally fitted (black lines) to two-state conformation change (FimHwt) [32] (A) and a 1:1 Langmuir (FimHFocH) (B) binding models. (PDF) [file ppat.1009440.s002.pdf]

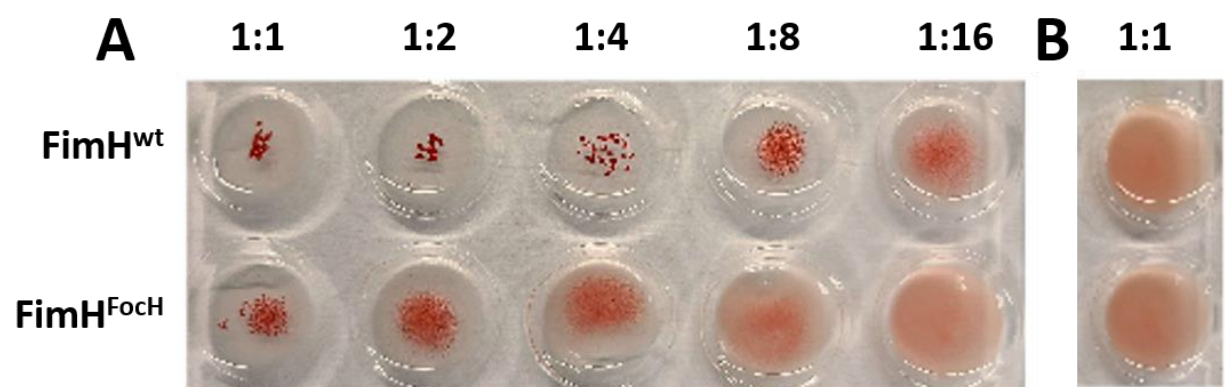

Supplement: S3 Fig — (A) RBC aggregation without pre-incubation with mAb824, with serial dilutions of bacteria (1:1 corresponds to the OD = 1.0); (B) RBC aggregation upon pre-incubation with mAb824; at the highest bacterial dose. (PDF) [file ppat.1009440.s003.pdf]

LAS

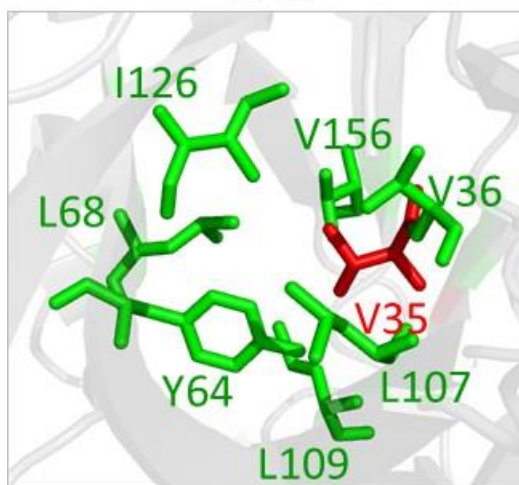

HAS

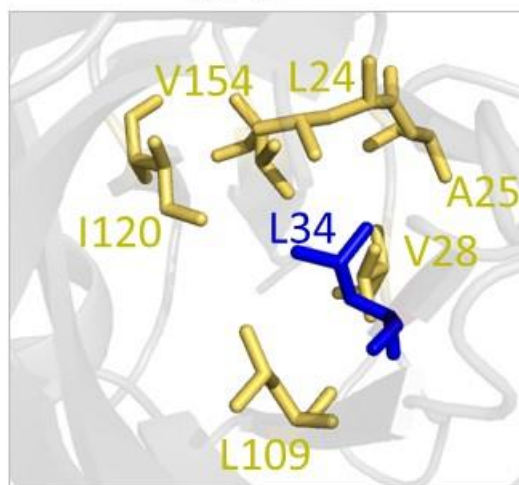

Supplement: S4 Fig — Altered network of residues in 3 Å distance (green and yellow) of core-buried V35 (left panel) and L34 (right panel) in LAS and HAS, respectively, are shown. The V35-interacting residues are: V36, Y64, L68, L107, L109, I126, V156, and L34-interacting residues are: L24, A25, V28, L109, I120, V154. (PDF) [file ppat.1009440.s004.pdf]

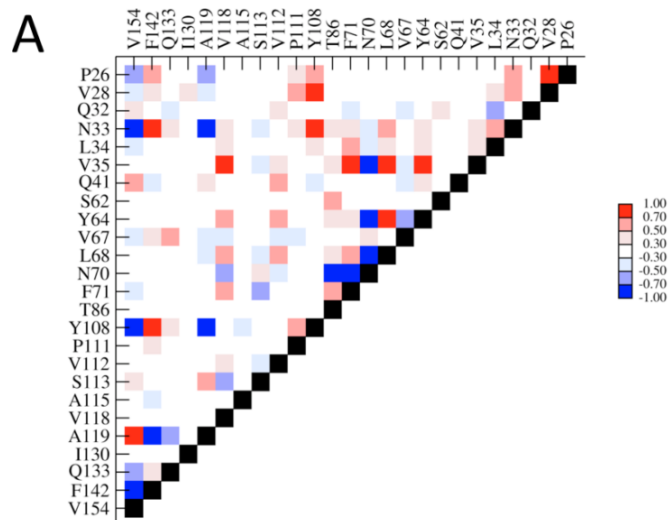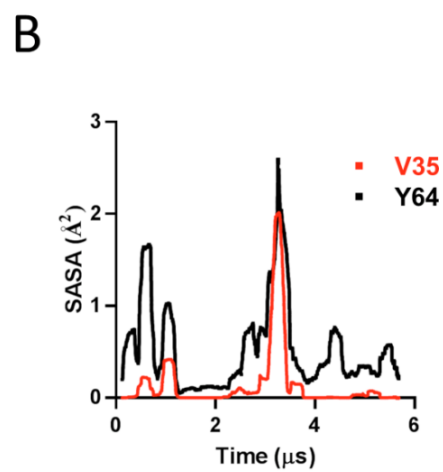

Supplement: S5 Fig — (A) A previously published MD simulation [20] performed at 330 K on the Anton supercomputer, a resource dedicated to the production of long MD trajectories, was re-analyzed to study correlations between time series of SASA values of side chains that are observed to change between surface and core orientations when comparing X-ray structures of LAS and HAS. A total of 25 side chains were compared pairwise and the Pearson’s linear correlation coefficient was calculated between the 240-ns time averages of their SASA values using the program xmgrace. Color scale indicates the strength of the correlation. (B) Time course of the solvent accessibility of V35 and Y64 along the 5.8-μs long MD simulation [20]. Plotted are running averages over a time window of 240 ns. (PDF) [file ppat.1009440.s005.pdf]

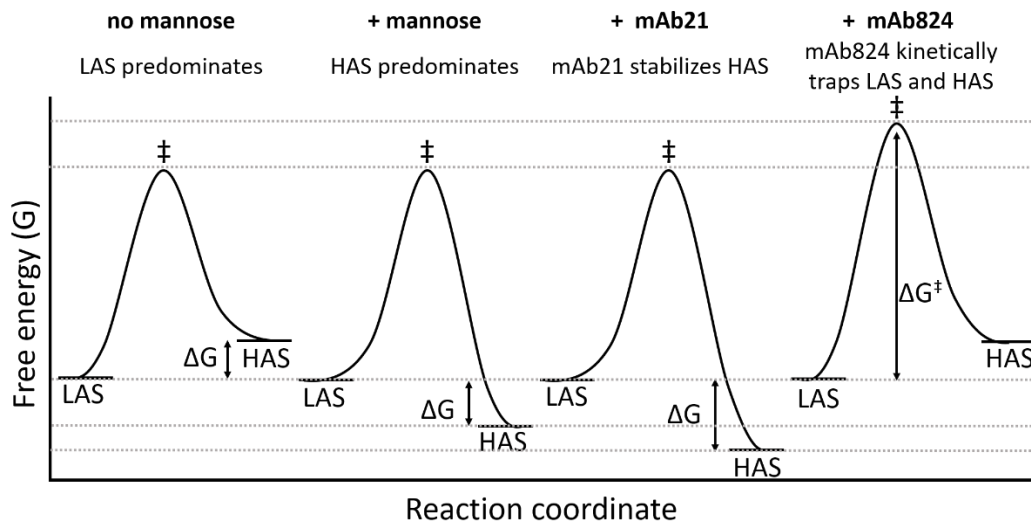

Supplement: S7 Fig — In the absence of mannose, LAS predominates while HAS predominates in the presence of mannose. mAb21 binding further stabilizes HAS. mAb824 traps both HAS and LAS by increasing the transition-state free energy (ΔG‡) required to transit between the two states, lowering the frequency of the switch in both directions. (PDF) [file ppat.1009440.s007.pdf]
