# Supplementary material for: Toggle switch residues control allosteric transitions in bacterial adhesins by participating in a concerted repacking of the protein core
Source: PLoS Pathog. 2021 Apr 7;17(4):e1009440. doi: 10.1371/journal.ppat.1009440 (PMC8064603; doi:10.1371/journal.ppat.1009440)
Supplement: S6 Fig — The identity/similarity of sequences is marked by red/grey shading. Sequence numbering is the same as in S4 Table. L34, V35 and Y64 positions in FimHwt (sequence #1) are marked by asterisks. (PDF) [file ppat.1009440.s006.pdf]

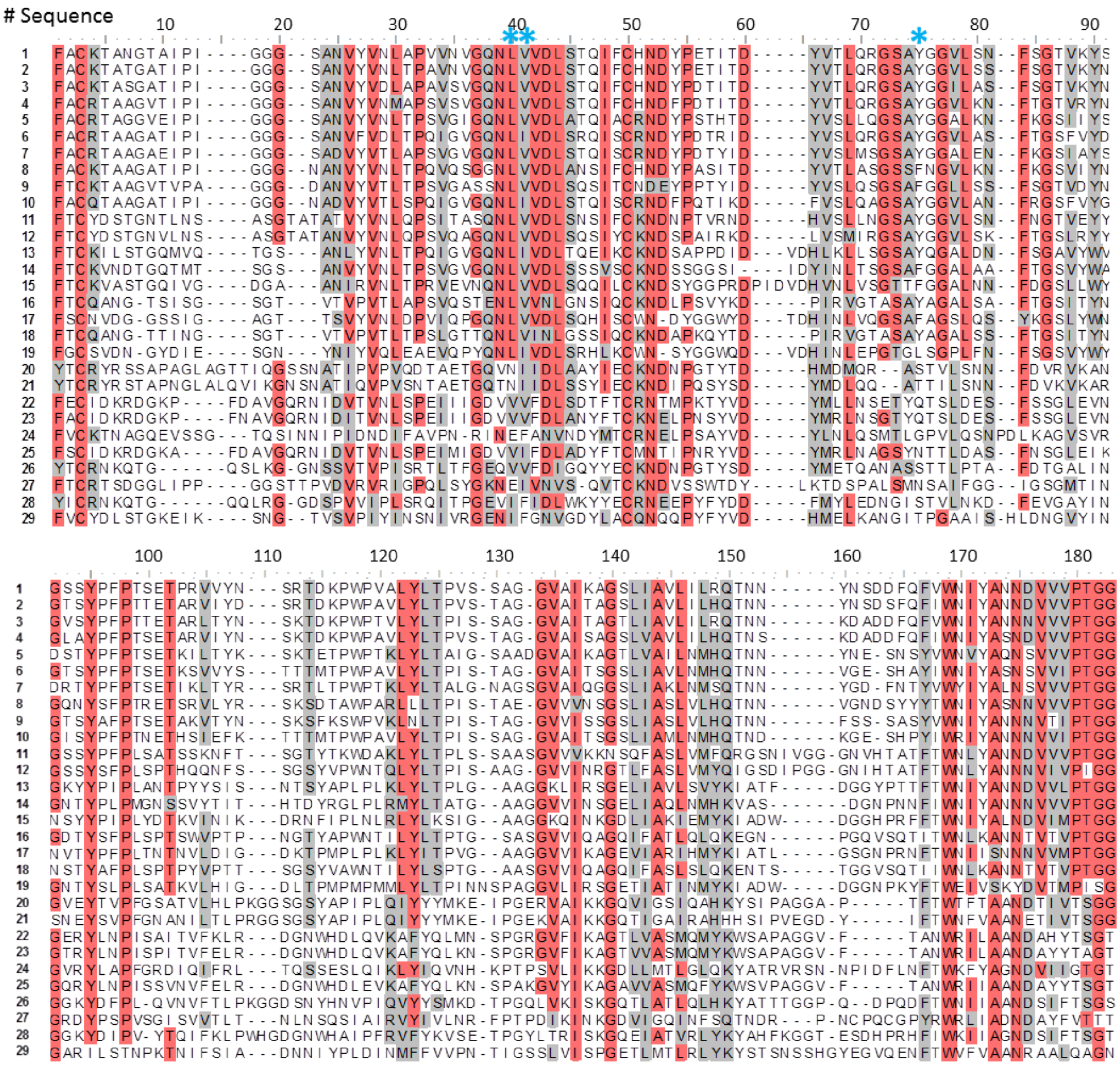

Sequence
