# Supplementary material for: Toggle switch residues control allosteric transitions in bacterial adhesins by participating in a concerted repacking of the protein core
Source: PLoS Pathog. 2021 Apr 7;17(4):e1009440. doi: 10.1371/journal.ppat.1009440 (PMC8064603; doi:10.1371/journal.ppat.1009440)
Supplement: S1 Table — (PDF) [file ppat.1009440.s008.pdf]

	mAb824*			mAb824			mAb824	
**Mutation**	**FimH^{wt}**	**FimH^{FocH}**	**Mutation**	**FimH^{wt}**	**FimH^{FocH}**	**Mutation**	**FimH^{wt}**	**FimH^{FocH}**
**F1L**	99	100	**T57R**	100	95	**T90N**	94	86
**A2S**	105	NT	**Q59A**	97	NT	**P91A**	93	84
**G8A**	103	NT	**R60P**	104	77	**P91R**	3	5
**T9G**	89	NT	**Y64F**	99	NT	**R92A**	100	105
**A10P**	102	NT	**Y64L**	90	NT	**R92D**	105	NT
**P12A**	108	101	**Y64E**	82	NT	**V93A**	100	82
**I13S**	99	NT	**Y64A**	81	NT	**P104L**	101	91
**N19C**	99	NT	**Y64R**	90	NT	**P104W**	101	101
**L24A**	NT	93	**V67K**	103	98	**A106V**	100	NT
**A25V**	101	NT	**L68V**	91	NT	**A106E**	90	NT
**P26G**	96	109	**S69C**	65	NT	**Y108E**	104	NT
**P26A**	92	93	**N70C**	88	NT	**P111K**	NT	101
**P26E**	99	96	**N70G**	92	NT	**S113C**	96	NT
**P26H**	90	NT	**F71A**	104	NT	**S114C**	95	NT
**V27A**	86	99	**S72A**	98	96	**G116C**	102	NT
**V27W**	105	90	**G73A**	100	104	**G117D**	85	NT
**V28T**	NT	94	**T74A**	92	88	**K121G**	104	NT
**N29C**	100	NT	**K76A**	113	99	**K121D**	93	NT
**V30C**	NT	109	**Y77A**	NT	100	**A122H**	104	NT
**N33L**	100	NT	**Y77N**	NT	92	**S124A**	98	NT
**N33K**	89	NT	**S78A**	105	98	**I130A**	92	NT
**L34A**	105	NT	**S78Y**	98	90	**R132D**	100	98
**L34E**	100	NT	**S78W**	NT	88	**R132H**	NT	106
**L34V**	103	NT	**G79A**	109	88	**Q133N**	89	94
**L34K**	94	98	**G79R**	4	6	**Q133A**	NT	97
**V35A**	109	NT	**S80A**	47	5	**T134G**	NT	98
**V35E**	88	NT	**S80R**	3	8	**N135I**	96	99
**V35F**	104	NT	**S80T**	106	NT	**N136A**	NT	89
**V35L**	105	NT	**S81A**	106	107	**Y137A**	103	99
**N46Q**	101	99	**S81G**	95	103	**N138I**	101	95
**N46A**	100	NT	**S81R**	96	14	**S139A**	NT	99
**D47S**	NT	97	**Y82A**	4	40	**F142A**	93	NT
**Y48A**	96	NT	**P83S**	97	100	**Q143A**	96	96
**E50A**	105	103	**P83R**	96	91	**V145I**	107	NT
**T51A**	107	98	**F84S**	96	80	**A150V**	103	98
**I52A**	101	98	**P85S**	98	109	**V155H**	84	NT
**T53A**	88	NT	**T87A**	106	95	**V155W**	NT	90
**T53R**	103	NT	**E89A**	65	94	**V156P**	NT	93
**D54E**	NT	81	**E89K**	99	NT			
**Y55A**	NT	98	**T90G**	99	98			

*Binding of mAb824 to purified isogenic fimbriae with different mutations in the lectin domain (LD) of FimH^{wt} and FimH^{FocH}. Relative binding (%) of the antibody as compared to not mutated LD of FimH^{FocH} is shown. The mutations reducing mAb binding >50% are marked in red. NT, not tested.
