# Supplementary material for: Toggle switch residues control allosteric transitions in bacterial adhesins by participating in a concerted repacking of the protein core
Source: PLoS Pathog. 2021 Apr 7;17(4):e1009440. doi: 10.1371/journal.ppat.1009440 (PMC8064603; doi:10.1371/journal.ppat.1009440)
Supplement: S2 Table — (PDF) [file ppat.1009440.s009.pdf]

SASA*								
FimH^{wt} AA	LAS (4xo9)	HAS (4xo8)	FimH^{wt} AA	LAS (4xo9)	HAS (4xo8)	FimH^{wt} AA	LAS (4xo9)	HAS (4xo8)
PHE1	263	16	ASP54	9	5	LEU107	0	1
ALA2	28	15	TYR55	31	54	TYR108	14	63
CYS3	9	5	VAL56	0	0	LEU109	0	1
LYS4	80	73	THR57	14	6	THR110	61	38
THR5	12	7	LEU58	0	0	PRO111	53	10
ALA6	57	68	GLN59	30	45	VAL112	109	42
ASN7	126	115	ARG60	144	114	SER113	52	115
GLY8	44	50	GLY61	45	2	SER114	139	102
THR9	74	57	SER62	0	34	ALA115	96	10
ALA10	75	67	ALA63	23	9	GLY116	29	38
ILE11	12	2	TYR64	13	88	GLY117	49	27
PRO12	96	80	GLY65	5	44	VAL118	28	77
ILE13	64	119	GLY66	42	13	ALA119	69	3
GLY14	25	34	VAL67	86	6	ILE120	1	1
GLY15	76	17	LEU68	0	105	LYS121	123	141
GLY16	54	33	SER69	82	80	ALA122	53	57
SER17	78	79	ASN70	147	35	GLY123	44	54
ALA18	6	6	PHE71	37	16	SER124	39	29
ASN19	86	60	SER72	61	63	LEU125	43	48
VAL20	3	3	GLY73	11	18	ILE126	0	2
TYR21	106	119	THR74	26	49	ALA127	0	0
VAL22	0	8	VAL75	0	0	VAL128	42	23
ASN23	68	125	LYS76	79	79	LEU129	0	0
LEU24	10	12	TYR77	2	6	ILE130	34	13
ALA25	69	46	SER78	63	73	LEU131	0	0
vPRO26	22	84	GLY79	69	68	ARG132	66	65
VAL27	68	83	SER80	52	53	GLN133	44	6
VAL28	103	3	SER81	77	71	THR134	37	32
ASN29	71	61	TYR82	55	60	ASN135	27	23
VAL30	148	79	PRO83	75	73	ASN136	83	86
GLY31	64	73	PHE84	4	10	TYR137	127	134
GLN32	43	88	PRO85	55	61	ASN138	62	69
ASN33	5	94	THR86	7	32	SER139	108	126
LEU34	26	1	THR87	79	115	ASP140	68	50
VAL35	0	70	SER88	64	69	ASP141	64	78
VAL36	0	5	GLU89	70	76	PHE142	133	51
ASP37	55	59	THR90	10	12	GLN143	117	83
LEU38	0	0	PRO91	69	77	PHE144	8	0
SER39	31	36	ARG92	133	162	VAL145	43	34
THR40	70	71	VAL93	14	17	TRP146	1	0
GLN41	20	46	VAL94	63	57	ASN147	24	33
ILE42	0	0	TYR95	6	3	ILE148	0	0
PHE43	50	48	ASN96	101	99	TYR149	42	61
CYS44	0	0	SER97	36	45	ALA150	1	4

**HIS45**	36	37	**ARG98**	125	115	**ASN151**	77	76
**ASN46**	2	2	**THR99**	80	100	**ASN152**	43	38
**ASP47**	68	61	**ASP100**	41	70	**ASP153**	87	70
**TYR48**	140	105	**LYS101**	103	70	**VAL154**	21	0
**PRO49**	37	40	**PRO102**	58	56	**VAL155**	91	55
**GLU50**	128	124	**TRP103**	0	0	**VAL156**	0	1
**THR51**	103	71	**PRO104**	37	33	**PRO157**	25	52
**ILE52**	32	22	**VAL105**	**2**	**0**	**THR158**	14	175
**THR53**	28	38	**ALA106**	7	11			

*The solvent accessible surface area (SASA) of amino acid residues in LAS and HAS conformers (PDB codes 4xo9 and 4xo8, respectively) were calculated individually by CCP4 software (see Materials and Methods). The amino acid residues of the functional epitopes were marked in red and the amino acid residues that showed large signal perturbation in NMR are shown in blue.
