# Supplementary material for: Toggle switch residues control allosteric transitions in bacterial adhesins by participating in a concerted repacking of the protein core
Source: PLoS Pathog. 2021 Apr 7;17(4):e1009440. doi: 10.1371/journal.ppat.1009440 (PMC8064603; doi:10.1371/journal.ppat.1009440)
Supplement: S3 Table — (PDF) [file ppat.1009440.s010.pdf]

Methyl Peaks Residue #	Assigned (A) Unassigned (U)	NMR observations Large effects (L) Small effect /Not perturbed (S)
A2	A	S
T5	A	S
A6	A	S
T9	A	S
A10	A	S
I11	A	L
I13	A	S
A18	A	S
V20	U	
V22	A	S
L24	A	L
A25	A	S
V27	U	
V28	U	
V30	U	
L34	A	L
V35	A	L
V36	A	L
L38	A	L
T40	U	
I42	A	L
T51	U	
I52	A	L
T53	A	S
V56	A	L
T57	A	S
L58	A	L
A63	U	
V67	A	L
L68	A	S
T74	U	
V75	A	L
T86	U	
T87	U	
T90	A	L
V93	A	
V94	U	
T99	A	
V105	A	L
A106	A	L
L107	A	L
L109	A	L
T110	A	S
V112	A	S

A115	A	S
A118	U	
A119	A	S
I120	A	L
A122	A	S
L125	A	L
I126	A	L
A127	A	S
V128	U	
L129	A	L
I130	A	S
L131	A	L
L134	U	
V145	U	
I148	A	L
A150	A	L
V154	U	S
V155	U	
V156	A	S
T158	U	
