# Supplementary material for: Toggle switch residues control allosteric transitions in bacterial adhesins by participating in a concerted repacking of the protein core
Source: PLoS Pathog. 2021 Apr 7;17(4):e1009440. doi: 10.1371/journal.ppat.1009440 (PMC8064603; doi:10.1371/journal.ppat.1009440)
Supplement: S4 Table — (PDF) [file ppat.1009440.s011.pdf]

Number	Accession number*	Species	Identity (%)†	Receptor specificity	Protein Length (AA)
1	P08191	*Escherichia coli*	query	α-D-mannose	300
2	A6TDM0	*Klebsiella pneumoniae*	88	α-D-mannose	301
3	A0A2X3GE33	*Kluyvera cryocrescens*	84		301
4	A0A0L0AF88	*Klebsiella sp.*	81		301
5	B2VEY7	*Erwinia tasmaniensis*	67		304
6	U2MA57	*Serratia fonticola*	66		305
7	A0A1I4X1B9	*Izhakiella capsodis*	66		313
8	A0A0J8YTF1	*bacteria symbiont BFo2 of Frankliniella*	64		301
9	A0A085JND3	*Tatumella ptyseos*	63		187
10	W0LFI5	*Chania*	63		305
11	W0L8D6	*Chania*	54		311
12	A0A1B7JTX7	*Providencia heimbachae*	52		312
13	A0A0J5NKQ8	*Pluralibacter gergoviae*	46		302
14	A0A336Q1W3	*Citrobacter koseri*	43		300
15	B4F2K7	*Proteus mirabilis*	42		310
16	A0A095UYJ3	*Tatumella morbirosei*	41		299
17	P77588	*Escherichia coli*	41	Gal(β1-3)GalNac	304
18	A0A085JP12	*Tatumella ptyseos*	41		191
19	B4ETK5	*Proteus mirabilis*	34		303
20	A0A348HGN6	*Zymobacter palmae*	28		304
21	A0A348HGN5	*Zymobacter palmae*	28		337
22	A0A1E7Z0K3	*Candidatus Erwinia*	27		309
23	A0A0J8YKK0	*bacteria symbiont BFo1 of Frankliniella* [illegible]	26		311
24	K8WLZ7	*Providencia sneebia*	25		333
25	A0A0L7THN9	*Erwinia iniecta*	24		326
26	B2VGL4	*Erwinia tasmaniensis*	24		323
27	D4DZU5	*Serratia odorifera*	24		310
28	A0A2G8E182	*Erwinia sp.*	19		264
29	A0A495RCD0	*Orbus hercynius*	18		321

*Selected structural homologues of FimH lectin domain as determined by Pfam database search using the sequence of FimH^{wt} (P081191) as query

†Identity (%) of lectin domain sequence (1-160 AA) is shown
