# Supplementary material for: Toggle switch residues control allosteric transitions in bacterial adhesins by participating in a concerted repacking of the protein core
Source: PLoS Pathog. 2021 Apr 7;17(4):e1009440. doi: 10.1371/journal.ppat.1009440 (PMC8064603; doi:10.1371/journal.ppat.1009440)
Supplement: S1 Movie — During this simulation time frame, the flipping of residues L34 (at 4 seconds in the movie) and V35 (at 37 seconds) was observed. L34 and V35 are colored in blue and red, respectively, and their side chains are shown in the stick and ball representation and labeled. Distances between atoms involved in backbone hydrogen bonds between V36 and L107 are indicated by blue dashed lines. Atoms involved in the V36 NH… O L107 hydrogen bond are colored in black while those in the L107 NH… O V36 hydrogen bond are colored in green, respectively. (PDF) [file ppat.1009440.s012.pdf]

S1 Movie
