# Supplementary material for: Toggle switch residues control allosteric transitions in bacterial adhesins by participating in a concerted repacking of the protein core
Source: PLoS Pathog. 2021 Apr 7;17(4):e1009440. doi: 10.1371/journal.ppat.1009440 (PMC8064603; doi:10.1371/journal.ppat.1009440)
Supplement: Attachment — Submitted filename: Response to reviewers.docx [file ppat.1009440.s014.docx]

Answers (As) to the Reviewer's comments (Cs):

Reviewer #1:

C1. No additional scientific experiments are needed. However, there are figures that are confusing and/or do not show what the authors are hoping to convey. These are discussed below.

*A1: We re-worked the figures extensively to make them clearer and more informative.*

Reviewer #2:

C2. The authors have made no attempt to explain why their binding data disagree with other molecular explanations of the FimH catch-bond mechanism. It is my understanding that it was thought that ligand (mannose) binding does not lead to the separation of the pilin and …. that this transition only occurs in the presence of tensile forces. My question is simply how do the authors explain that mannose titration leads to domain separation and interaction with mAb21? …. Or were these experiments conducted under shaking conditions? If not, how does this fit in with existing data? Experiment suggestion: could you use NMR to directly show that mannose binding leads to domain separation? To validate that mAb21 binding actually reports on transition to the high-affinity domain separated state.

*A2. We have expanded the discussion on the excellent 2016 Sauer et al. study* (1) *which suggested that tensile force is required for domain separation and, therefore, LAS to HAS transition. We have added a depiction of the intermediate (hybrid) FimH state identified in that study into Figure 1A. We also reference their more recent study* (2) *which showed that mannose binding destabilizes the interdomain interaction under equilibrium conditions (without mechanical force applied) thereby enabling domain separation, albeit with relatively slow kinetics. Based on this observation, the authors of the 2019 study* (2) *stated the validity of our previous observations that HAS-specific monoclonal antibodies, like mab21, that bind to FimH in the presence of mannose stabilize the HAS conformation.*

*Thus, there is no basic disagreement between the models previously proposed by us and that in the Sauer et al. studies. Both conform to the allosteric model of catch bonds in which formation of a strong FimH-mannose bond requires both binding of the ligand AND domain separation. While we proposed that mannose alone can cause the LAS to HAS conversion of the lectin domain (LD), we also emphasized that tensile force is expected to contribute by facilitating and, importantly, maintaining domain separation under flow. Thus, mannose binding and domain separation are reciprocal allosteric events, one facilitating another. The tensile force-induced domain separation is likely critical when the transition must happen fast to ensure strong bond formation under dynamic flow (shaking) conditions. The mannose-induced transition is likely slower and plays a role during adhesion under static or equilibrium conditions. In both cases, however, the allosteric propagation across the LD, either from the binding pocket to the interdomain interface or in the reverse direction, involves same basic molecular mechanism. We expand and clarify this important discussion in the revised manuscript.*

*The reviewer asks whether it’s possible to determine whether mannose can separate the domains using NMR. Unfortunately, this is not possible for technical reasons, as the size of FimH is not conducive to detailed NMR investigation. Instead, we have included additional data in Figure 1 (now panel D) to show that FimH-mAb21 complex has HAS-like activity under equilibrium conditions, consistent with the notion that mannose alone is able to induce HAS.*

C3: Why have the authors chosen to use the FimHFocH construct as the proxy for the HAS? Would it make sense to also include lectin domain only constructs as a further control in the binding experiments? I was struck by the discrepancy in the reported KD differences between wt and FimH-FocH (~10 fold) compared to the affinity differences previously reported (>1000 fold) for the low and high affinity states using different constructs.

*A3: We clarify this issue by noting previous studies where we have shown that LD interaction with the chimeric FocH-like pilin domain is markedly altered so that the HAS is the predominant state of LD in FimHFocH (based on its similar binding affinities as the purified LD and, also, the monoclonal antibodies recognition). Our use of fimbria-incorporated full-size FimH across the study allows a more direct functional comparison of different structural variants of FimH in the antibody and mannose binding assays [ref 7].*

*We clarify in the discussion that the smaller difference in the affinity for RNAseB between native FimH and FimH trapped in HAS is likely because RNAse B has abundant Man5 oligosaccharide moieties. Among the natural ligands, Man5 has the highest affinity to LAS conformers of FimH due to the presence of terminal oligomannose in Man(a1-3)Man(b1-4)(GlcNAc)2-Asn where interaction with the terminal mannosyl is stabilized with additional interactions between the internal saccharides and FimH residues outside the binding pocket itself* (3)*. More significant affinity differences reported previously by us and other groups were based on measurements done with either mannose derivatives or mannosylated oligo-saccharides without the terminal oligosaccharide. Those ligands interact with FimH via the mono-mannose mechanism that results in a strong binding of FimH/LD in HAS but not in LAS conformation.*

C4: I recommend revising the terminology of “active” and “inactive” FimH conformations used throughout the manuscript. In my view, the low and high-affinity states of FimH are not the same as inactive and active FimH.

*A4: We agree, but ‘active’/’inactive’ is the standard and broadly accepted terminology for different states of allosteric receptor proteins. So, we replaced ‘active’ with HAS and ‘inactive’ with LAS throughout the text, but indicated the terminology concordance in the abstract and introduction.*

**Reviewer #1:**

C5: The pilus type under study is not stated until line 410. It is important to say earlier that FimH is the adhesin from type 1 pili expressed on E coli, and more specifically, on UPEC.

*A5: We now state that we are focused on the FimH variant and fimbriae that are structurally identical to those expressed by the model uropathogenic strains J96 and are especially common among uropathogenic E. coli in the beginning of the Results section.*

C6: The NMR methodology is not described in a manner that would make the data reproducible. In particular, it would be helpful to include information on the 13C-methyl spectral analysis, or at least provide a reference to how these data are processed and analyzed.

*A6: To ensure that the NMR data can be reproduced by others, we have made additions to the materials and methods section. The NMR spectra were collected using standard parameters. In addition to the sample preparation and data processing we initially described, we have added that the data were visualized in NMRviewJ* (4) *and the NMR assignments for the FimH lectin domain were obtained from the Biological Magnetic Resonance Data Bank (BMRB).*

C7: With respect to figures, the introduction (starting at Line 78) would be improved by a figure. Perhaps simply a reference to Figure 1A would suffice.

*A7: As suggested, we reference the now expanded Figure 1A already in the introduction.*

C8: Looking at pdb's 3jwn, 1uwf and 6gtw, it is clear that the shape of the binding site goes from wide open to an almost fully enclosed cavity (see figure). this is not conveyed in the diagram, and it would be helpful if it could be somehow included. Also, I do not see a new large linker appearing between the subunits. Yes, the binding site for mAb21 becomes exposed, but the diagram seems to create an incorrect impression of the change.

*A8: The mannose-binding pocket in Figure 1A is now highlighted to make it clearer that it undergoes a shift from a wide-open to narrow-closed configuration. We also modified the linker configuration to avoid any misleading impression that the linker is significantly extended in HAS.*

C9: Figure 2D does not convey what the authors describe. It is not possible to see what is buried and what is exposed, nor understand what happens to L34 and V35 in the two states.

*A9: Figure 2D was modified to more clearly illustrate the core-surface positions of the toggle residues. To better illustrate buried versus solvent exposed residues, we have added stick representations (shown in gray) to depict amino acid residues that are buried in the protein core.*

C10: Figure 3 is not clear. For example, there are 2 labels for each of the residues L34, V35, and V36, L107. perhaps color all the labels to match the color of the signal (blue vs black)?

*A10: Leucine, valine, and isoleucine sidechains each have two methyl groups and these each give a peak in the spectrum. We make this clear in the figure caption and have altered the figure to clarify this.*

C11: Figure 4 does not show all the data needed to assess and understand the results. There should be a movie showing that at the specified time points L34 flips in and V35 flips out. This need to include movies is true to support all the MD data.

*A11: We have provided a Supplementary Movie showing the flipping of L34 and V35 in*

*the TMD simulation and have highlighted the time points of these events in Figure 4.*

C12: line 270. half turn rotation of the side chain. This is not clear in the figure 2D. What is the rotation axis?

*A12: Describing the side chain motion required to get from one end-state to the other is difficult because it involves changes in a number of torsion angles so finding a singular “rotation axis” is difficult. As it is the change in the sidechain’s buried versus exposed orientation that is important, we removed mention of rotations from the text.*

C13: line 284. how does fig 4A show beta strand unfolding before the L34 switch?

*A13: As mentioned in A11 above, we updated the figure to show the time points where L34 flips in and V35 flips out, respectively. We also modified the Figure 4 to more accurately describe the events observed and improve the text flow.*

C14: line 345. 13 residues are more solvent exposed and 16 are less exposed in the high affinity state. This goes against what is depicted in Fig 1A, so that figure is misleading, with its appearance of a new extended linker that would be exposed.

*A14: We provide more information on the SASA differences and indicate that the total SASA of LD in HAS is 7460 Å^2^, while total SASA of LD in LAS (including the interdomain interface) is 7559 Å^2^. Thus, there is indeed only 1.3% difference between them. Changes have been made to Fig 1A to show that the linker length is similar in HAS and LAS states.*

C15: Fig S4B label should be A119, not A1119.

*A15: Corrected.*

**Reviewer #2:**

C16: Line 78: Is it really the case that FimH is the most prevalent adhesin of E. coli? This is a very strong statement and I am not sure this is the case. Especially since chaperone-usher pilus expression (e.g. type 1 pili – FimH, P pili – PapG) is subject to complex regulation over the course of an infection.

*A16: We have added a reference supporting our statement that type 1 pili are indeed the most prevalent fimbriae type in E. coli* (5)*, with over 90% of strains being able to express them. Furthermore, when bacteria carry genes for other fimbriae (for example, genes for P pili are present in ~20-40%), their expression is cross-regulated with the type 1 fimbriae, so usually only one or another type is being expressed by a bacterial cell, depending on the conditions. We have modified the text to make this point more clearly.*

C17: Line 83: low affinity state does not equal inactive FimH (it still binds and this low-affinity binding is functionally relevant);

*A17: As suggested, we now use low- and high-affinity terminology only.*

C18: Line 93: typo – “catch bond-interactions”.

*A18: Corrected. Thank you.*

C19: Line 183: The off-rate is significantly reduced compared to what? This is not clear. There are no statistics provided, so the word “significant” should be avoided altogether.

*A19: We thank the reviewer for pointing out this typographical error. We intended to say that on-rate (not off-rate) of the antibody trapped in HAS was reduced relative to the non-trapped FimH-FocH, providing a possible explanation of the inhibitory effect of mab824. We have corrected this. Also, instead of ‘significantly’ we are saying substantially when describing the on-rate reduction. We also indicated that the standard error that was given in the table for the kinetic parameters relates to the “fit” of the data to the model.*

C20: Lines 244-246: What is the difference between “only a very small blue peak remaining in original position” and “loss of intensity”? Perhaps providing some example residues that exhibit these types of effects on the resonances in Fig 3 would be helpful for the reader.

*A20. For clarification, we now say ‘loss of intensity’ to refer to the peaks that have a “very small blue peak remaining at the original position”. We have highlighted some examples of peaks with intensity loss with red boxes.*

C21: Lines 257-258: Is this really the case? Does Fig 2 not indicate a gap between patches?

*A21: We clarify that there are no unperturbed methyl-containing surface-exposed residues between the patches, the presence of which would be contrary to our hypothesis that the two patches are part of a continuous structural epitope of mab824.*

C22: Line 262: There appear to be many more NMR-perturbed residues based on Fig 3? Which criteria were applied to choose NMR-perturbed residues? Please specify in the methods section.

*A22: We clarify that T90, L24, L34, V35, and A106 are the only surface-exposed methyl-containing residues that have a perturbed NMR spectrum. A full list of perturbed residues, both surface-exposed and core-buried is in Supplemental Table 3.*

C23: Line 322: Words missing? And “not due” to a misfolded form. What control experiments were conducted to rule this out?

*A23: The reviewer is correct – “not due” is missing. Moreover, the corresponding sentence was supposed to follow the next sentence, which says that the mab21 epitope is formed in the L34E LD, when the latter is fused to the non-interacting FocH pilin domain, i.e. identically to what is observed with the native, wild-type LD. In our opinion, this is an indication that the L34E LD is unlikely to be a misfolded protein. We hope the wording is clearer now.*

C24: Please discuss how these results fit in with the other models of the catch-bond e.g. where mannose binding does not yet lead to full domain separation (perhaps begins to shift the equilibrium), and that the high-affinity state only becomes prevalent under tensile force conditions that separate the PD and LD.

*A24: As mentioned in A2, we have expanded the discussion of the role of tensile force and mannose binding in the domain separation.*

C25: Table 1 There are two identical kconf rows for both FimH wt and FimH-FocH? Copy-paste error?

*A25: Thanks for catching this error. The second one should have read k-conf for both structures.*

C26. Please explain why you used two different equations for the KD depending on the FimH construct and define what kconf and k-conf are – perhaps in the Methods section.

*A26: We have now explained this in the methods as follows: Because the response for FimHFocH could be fit with a 1:1 binding model, this model was used for that data. Because the 1:1 model could not fit the FimHwt data, a more complex conformational change model, used previously to model native, catch-bond forming FimH variants* (6) *was used for that data. This is consistent with the notion that HAS is predominant in FimHFocH, while FimHwt is in equilibrium between HAS and LAS conformations.*

C26: Were the RBC agglutination results dose dependent? Such a result would be much more convincing if one could see raw data and not just the time upon which noticeable aggregation was observed.

*A26: We obtained results on the dose dependence and now provide the raw data in the Supplement. While the RBC agglutination is dose dependent, the data correspond well to the timing of RBC agglutination and the size of aggregates formed reflects the ability of FimH to form bonds under dynamic conditions.*

C27: Figure 2. What is the explanation for why the mutagenesis-identified patch is not the same as the patch identified by NMR? Or at least overlapping?

*A27: We clarify that the mutagenesis patch is composed of residues that do not have methyl residues and thus are not reported on in the methyl TROSY NMR spectrum.*

C28: Figure 3. There seem to be many more residues perturbed by mAb824 binding than mentioned in the text?

*A28: As mentioned in A22, the issue is that the perturbed buried residues are not visible in the surface renditions, but a complete list of perturbed residues is listed in the Supplemental Table 3. To avoid confusion, we emphasize this point more now.*

C29: Figure 5. The y-axis scale is quite different for these experiments compared to those in Fig 1.

*A29: The difference is likely because the experiments reported in Figure 1 and 5 were performed on separate days and different batches of the ELISA TMB substrate were used. We clarify in methods that all key comparative studies of different mutants or other conditions were always performed in parallel to each other, using same batch of antibodies or ELISA substrate, etc.*

C30: Methods. Is it sensogram or sensorgram?

*A30: It should be sensorgram and any typos found were fixed.*

**Reviewer #3: Specific comments:**

C31: Line 119: ‘αmm, hereinafter called mannose’ This is a bit confusing, since ‘amm’ is used in the text.

*A31: We now use αmm throughout the manuscript.*

C32: Line: 201: It would be helpful if the rationale of the selection of residues for mutagenesis is explained.

*A32: We clarify in the Methods that we used an extensive library of FimH mutants compiled in multiple previous studies as the basis for our selections. In cases when candidate epitope residues were found, more targeted mutagenesis was performed.*

C33: Line 577: ‘Gram-negative bacterial…’

*A33: Corrected the ‘gram-‘ to ‘Gram-’.*

C34: Figure 2, panels B and C. The arrows showing the rotation between the views should point in the opposite direction.

*A34: Corrected. Thank you.*

C35: Figure S4. This Figure contains an informative illustration of the structural transition and perhaps could be incorporated in the main text (just a suggestion).

*A35: The figure was moved to the main text as suggested.*

References:

1.	Sauer MM, Jakob RP, Eras J, Baday S, Eriş D, Navarra G, et al. Catch-bond mechanism of the bacterial adhesin FimH. Nat Commun. 2016 Mar 7;7:10738.

2.	Sauer MM, Jakob RP, Luber T, Canonica F, Navarra G, Ernst B, et al. Binding of the Bacterial Adhesin FimH to Its Natural, Multivalent High-Mannose Type Glycan Targets. J Am Chem Soc. 2019 Jan 16;141(2):936–44.

3.	Wellens A, Garofalo C, Nguyen H, Van Gerven N, Slättegård R, Hernalsteens J-P, et al. Intervening with Urinary Tract Infections Using Anti-Adhesives Based on the Crystal Structure of the FimH–Oligomannose-3 Complex. PLoS ONE [Internet]. 2008 Apr 30 [cited 2021 Feb 2];3(4). Available from: https://www.ncbi.nlm.nih.gov/pmc/articles/PMC2323111/

4.	Johnson BA, Blevins RA. NMR View: A computer program for the visualization and analysis of NMR data. J Biomol NMR. 1994 Sep;4(5):603–14.

5.	Johnson JR. Virulence factors in Escherichia coli urinary tract infection. Clin Microbiol Rev. 1991 Jan;4(1):80–128.

6.	Yakovenko O, Tchesnokova V, Sokurenko EV, Thomas WE. Inactive conformation enhances binding function in physiological conditions. Proc Natl Acad Sci U S A. 2015 Aug 11;112(32):9884–9.
